# Not All Selfies Took Alike: Distinct Selfie Motivations Are Related to Different Personality Characteristics

**DOI:** 10.3389/fpsyg.2017.00842

**Published:** 2017-05-26

**Authors:** Shir Etgar, Yair Amichai-Hamburger

**Affiliations:** The Research Center for Internet Psychology, Sammy Ofer School of Communication, Interdisciplinary Center HerzliyaHerzliya, Israel

**Keywords:** selfie, motivation, selfie motivation, personality, big five, narcissism, self-esteem

## Abstract

Selfies have become a frequent and commonplace occurrence, though the reasons which lead people to take selfies remain unclear. This research explores what motivates selfie taking, and suggests that this is not a uniform phenomenon and varying motivations may be found among selfie takers. In addition, the connection between these distinct selfie motivations and personality characteristics, including the big five, narcissism, and self-esteem, as well as types of selfie behaviors are examined. At the first stage of the research, 117 participants filled out a questionnaire dealing with their reasons for taking selfies. An explanatory factor analysis revealed three distinct selfie motivations: self-approval, belonging, and documentation. At the second stage, 191 different participants answered both the same questionnaire, and personality traits questionnaires. A confirmatory factor analysis verified that the three selfie motivations model has a good fit. Our results suggested that each selfie motivator is differently related to personality characteristics: self-approval was negatively related to: conscientiousness, emotional stability, openness to experiences, and self-esteem, and positively correlated to frequent checking for “likes.” Belonging was related to openness to experiences. Documentation was related to agreeableness and extroversion. Unlike previous studies, none of the selfie motivating factors was found to relate to narcissism. The reasons for these differences, as well as the need to refer to selfie taking as a multidimensional phenomenon, are discussed.

## Introduction

A “selfie” is a self-photograph, usually taken by cellphone or webcam, mainly in order to upload it to social network sites (SNS) (Weiser, 2015). The phenomenon of selfies is so common, that in 2013, the Oxford dictionary declared it is the “word of the year” (Oxford dictionaries, 2013). In 2015, Instagram hosted 238 million photos with a selfie hashtag (Weiser, 2015), a number that has now risen to 267 million. This indicates that in the space of about a year, 29 million selfies were uploaded to Instagram. Katz and Crocker (2015) surveyed and interviewed people from the United States, United Kingdom, and China about their selfie habits. Their results showed that within the age of 18–24, with no cultural differences, between 96 and 100% of participants reported having taken selfies. Most of participants also reported frequently upload selfies to SNS, however, its notable to mention that due to self-appearance worries and ideas about privacy, most of the selfies were not uploaded to SNS (Katz and Crocker, 2015). All these statistics suggest that selfie taking is a popular, common, cross-cultural phenomenon.

Thus, it is no wonder that in the recent years, the increasing number of selfies has prompted a growing interest as to the reasons people take and post selfies. Much of this research has implied that narcissism plays a role in selfie behaviors: For example, narcissistic individuals tend to take more selfies than non-narcissistic ones (Halpern et al., 2016), and to like posting selfies to SNS more than less-narcissistic individuals (Lee and Sung, 2016). Another study found that narcissism in general, and grandiose exhibitionism facets specifically, are related to the frequency of selfie posting (Weiser, 2015). Other study showed that vulnerable narcissism, the kind of narcissism in which an individual bases his or her self-esteem on others’ opinions, is found to be related to selfie posting (Barry et al., 2015). However, another study suggested that the relationship between selfie posting and narcissism is valid only for men (Sorokowski et al., 2015). An interesting piece of research asked participants directly, in an open question, whether selfie posting is encourage them to engage in narcissist behaviors, and more than 50% of the answers made a connection between taking a selfie and narcissistic behavior (Wickel, 2015).

In addition to narcissism, other explanations to selfie behaviors have been posited. One of them, suggested in a study in which participants were asked to fill a self-report survey regarding selfie taking and sharing during travels, is that a traveling selfie can be posted to SNS as a “real time” update, directed to others that are not sharing the same experience with you (Paris and Pietschnig, 2015). Similar ideas were also found in Warfield’s (2014) interviews with young women, from which she concluded that taking selfies helped them to sense “authentic.” All these findings were supported by Katz and Crocker (2015) survey and interviews, who demonstrated that people take selfies in order to control their self-presentation and identification, to prove they took part in an experience or event, and to receive feedback from their peers.

As Katz and Crocker (2015) concluded, “the selfie category encompasses a range of use and intention” (p. 1870). While most of the studies suggest there is one major reason to produce a selfie, Katz and Crocker advocate, there may in fact be a variety of motivations behind taking a selfie. This suggestion has not been given sufficient prominence, and with this in mind, our study aims to examine whether there might be several distinct motivations behind taking a selfie, since it might not be a unidimensional phenomenon as most studies suggest. Moreover, each of the studies mentioned above, described different, sometimes conflicting, motivations. For example, taking selfie for narcissist reasoning as suggested by Halpern et al. (2016), might perceived as the opposite of taking selfie to feel authentic, as suggested by Warfield (2014). These varied reasons may also be seen as a further indication that selfie taking is, as we suggest, a multidimensional phenomenon, and that several reasons to take a selfie may exist simultaneously. Thus, the current study will investigate how many selfie motivations exist, and what are these motivations actually are.

As mentioned earlier, selfies are directly connected to SNS, since some of them are being posted on SNS, and since they are a social phenomenon that begin and advance their power at SNS (Katz and Crocker, 2015). There is much evidence to show that behaviors displayed on SNS often relate to personality traits. For example, the compulsive use of SNS is positively related to extroversion, agreeableness, and neuroticism (Hsiao et al., 2017). Other research showed that Facebook users are perceived as being more extraverted and narcissistic as compared to non-users (Ryan and Xenos, 2011). Several studies found that higher Facebook use, number of Facebook friends, and amount of public information sharing are also positively related to extroversion (Ross et al., 2009; Amichai-Hamburger and Vinitzky, 2010; Seidman, 2013; Jain et al., 2016). In addition, neuroticism is negatively related to overall SNS use (Jain et al., 2016), but positively correlated to public sharing of personal information (Amichai-Hamburger and Vinitzky, 2010), and to sharing information about one’s ideal self on SNS (Seidman, 2013). Other personality characteristics were also related to SNS usage, for example, agreeableness was positively related to the amount of photo uploading, and negatively related to the amount of status updating (Amichai-Hamburger and Vinitzky, 2010; Jain et al., 2016), openness to experience was related to using a large number of Facebook tools, and conscientiousness was positively related to a greater amount of Facebook friends (Amichai-Hamburger and Vinitzky, 2010). The amount of updating of profiles were also related to higher self-esteem (Gonzales and Hancock, 2011). And it is not just the online behavior, even the topics that people chose to share on SNS were found to be related to their personalities: openness to change was positively related to sharing intellectual issues, extroversion was positively related to sharing information about social activities, higher self-esteem was related to less sharing about ones’ romantic experiences, and narcissism was related to sharing information about the self body image (Marshall et al., 2015).

The link between SNS and personality occurs on other sites as well: amount of YouTube usage was found to be related to extroversion and neuroticism, and amount of Instagram usage was related to all the big five personality traits (Hamid et al., 2015). Moreover, different Instagram behaviors, such as amount of time spent on Instagram and changing profile photos, as well as motivation to use Instagram, were related to narcissism (Moon et al., 2016; Sheldon and Bryant, 2016). With all this in mind, the next stage of our research was to examine the relationship between the selfie taking motivations and personality traits: in the case of divergent types of motives to selfie taking, it would seem logical to assume that they are related to different personality type.

There is evidence that selfie behaviors are related to personality traits. As mentioned earlier, most studies have focused on the selfie, as it relates to narcissism. However, Paris and Pietschnig (2015) found that positive attitudes toward selfies are positively related to emotionality and extroversion, while positive attitudes toward “travel selfies,” that are taken during a trip in order to share experiences, are positively related to agreeableness.

To explore the relationships between different selfie motivations and different personality traits, personality measures that were previously found to distinguish between SNS uses were used. For this study, the relationship between the particular selfie motivation that was found among subjects and the personality traits of narcissism, self-esteem, and the big five traits: Extraversion, agreeableness, conscientiousness, emotional stability, and openness to experiences was examined. We predicted that there are several motivations behind the act of taking a selfie, and each of these relates differently to personality characteristics.

Lastly, it is important to note that the term “selfie behaviors,” is used to denote both selfie taking and selfie posting to SNS. It seems that in the literature relating to selfies, the distinction between these two acts is not clear enough. For example, some studies discussed selfie taking, (for example, Warfield, 2014; Halpern et al., 2016), some talked about selfie posting (for example, Barry et al., 2015; Sorokowski et al., 2015; Weiser, 2015), and some dealt with both of these activities, (Katz and Crocker, 2015; Paris and Pietschnig, 2015). Overall, these terms were generally perceived as being similar to one another and sometimes used interchangeably. However, since only less than half of the selfies taken are in fact uploaded to SNS (Katz and Crocker, 2015), a distinction between taking and posting selfies is important. Selfie taking is a private act, while selfie posting is public, thus it arouses questions and concerns as to how others perceived the photo and what kind of feedback it will receive (Katz and Crocker, 2015). We believe that the time has come for a clear distinction to be made between these terms. This specific article will discuss only the motives of selfie taking, and their relationship to personality traits. Since selfie posting also involves the act of sharing the selfie with others, a different investigation should explore the motives behind selfie posting, and its connections to personality traits.

### The Current Study

Our research aimed to discover whether there are one or several motivations behind the taking of a selfie, and, in the case of several motivations, whether these are related to personality traits. In order to do this, we used a methodology that is similar to the one used by Paris and Pietschnig (2015). In Paris and Pietschnig’s (2015) study about taking and posting travel selfies, a pre-test in which participants freely write statements regarding their attitudes about travel selfies was conducted as a first stage. Based on this list, they produced an exploratory factor analysis (EFA), and based on that factor analysis, they examined which factor was related to each of the personality traits that they measured.

Similarly, we were first used a pre-test using the same free-writing assignment, in which participants freely indicated their motives behind selfie taking. We used their answers to produce an EFA to reveal the different selfie taking motives. At the second stage of the current research, an analysis was performed as to examine whether the factor structure that was found in the EFA can replicated in a new sample. The factor structure was tested using confirmatory factor analysis (CFA). If the factor’s pattern had, in fact, been replicated, the connections between the different factors and personality traits would be investigated.

## Materials and Methods

### Participants

In order to build and confirm different selfie motivations in two separate analyses, two stages of data were collected: At the first stage, 117 communications undergraduate volunteered to take part in the study. First stage sample included 82 women and 35 men, *M*_age_ = 23.07, *SD*_age_ = 1.81. For the second stage, 191 psychology undergraduates took part of the study in return to course credit. Of them, 128 were women and 63 were men. Unfortunately, due to technical problem, their age has not been recorded. However, as both stages were collected from undergraduates at the same collage, we assume that second stage participants were about the same age as first stage participants.

### Measures

#### Selfie Motivations

A pre-test was conducted on 11, volunteer, psychology undergraduates, all of whom were similar in age and gender distribution to the studies’ samples. Similarly to Paris and Pietschnig’s (2015) pre-test, participants were asked to write down as many motivations as they could think of to take a selfie.

They freely generated about 60 items. To avoid repetitiveness, items that were similar in meaning united into one item. As we wanted the motivation to represent the students’ state of mind accurately, we did not add any other item. We also did not delete any suggested item. That means that even items that appeared once during the pre-test were part of the selfie motivation questionnaire. Based on this pre-test, the selfie motivation questionnaire included 35 different types of motives to take a selfie. For example: “I’m taking selfie because it makes me feel less lonely,” or “I’m taking selfie because it helps me to meet new people,” or “I’m taking selfie because I want a souvenir from places that I visited.”

Participants were asked to indicate to which degree they agreed or disagreed with each sentence, on a 5-point Likert scale, ranged from 1-highly disagree, to 5-highly agree. To avoid repetitiveness, followed the instructions and before the list of different motives, it was written “*I’m taking a selfie because….*” Then, each sentence appeared without this beginning.

#### Selfie Behaviors

Participants were asked in which frequency they are uploading selfies to SNS, on a 4-point scale, ranged from 1 = “once a day and more” to 4 = “less than once a week.” Participants were also asked in which frequency they are checking the amount of likes their photo receives, on a 5-point scale ranging from 1 = “every minute” to 5 = “once a day or less.”

#### Self-esteem

Self-esteem was measured by the Rosenberg’s (1965) self-esteem scale, contains 10 items on a 4-point scale, ranged from “strongly agree” to “strongly disagree.” For example: “I take a positive attitude toward myself.” As in the original scale, answers were summed up, allowing a scale ranged from 10 to 40. The scale’s reliability was good at the current study (α = 0.88 at the second sample, in which we examined the relationship between self-esteem and selfie motivations).

#### Big Five

To examine the big five traits: Extraversion, agreeableness, conscientiousness, emotional stability, and openness to experiences, we used the Ten-Item Personality Inventory-(TIPI) (Gosling et al., 2003). This questionnaire assembled from 10 pairs of descriptions (for example, “Disorganized, careless”), and participants asked to rate the extent to which this pair of descriptions suitable to them, upon 7-point Likert scale, range from 1-strongly disagree to 7-strongly agree. Each of the big five traits is calculated based on the average of answers to two questions, one is a reversed version of the other (for example, agreeableness was calculated as the average of the score for “Sympathetic, warm,” and the reversed score for “Critical, quarrelsome”).

#### Narcissism

Narcissism was measured by the NPI-16 (Ames et al., 2006), a 16 items narcissism scale. Each item contains two sentences that are the opposite of each other, with a 5-points scale between them. Participants needed to choose the answer which they find as most identified of themselves. Choosing the middle item suggest a neutral opinion, while being more close to a sentence mean a larger agreement with it. For example, one of the items is “I am more capable than others,” against “There is a lot that I can learn from others.” Higher score among the scale referred to higher amount of narcissism. The scale’s reliability in the current study was good (α = 0.83 at the second sample, in which we will examine the relationship between self-esteem and selfie motivations).

### Procedure

In both samples, after agreeing to participate, participants were directed to a web questionnaire, and asked to sign a consent form. First they received general instructions about the survey and were informed that their anonymity would be preserved. Next, they answered the selfie behavior questions. Then, on a separate page, they filled the selfie motivation questions. Then, they answered the Rosenberg self-esteem questionnaire, the TIPI and the NPI-16. In the first sample, participants were then asked about their gender and age, while at the second sample, participants were asked about their gender at the beginning of the questionnaire. At the final screen, participants thanked for their participation. Both studies and the pre-test were conducted and run in Hebrew, and all the participants were native Hebrew speakers. It is also notable to mention that these studies were carried out in accordance with the recommendation of the APA ethical principles, Interdisciplinary Center Herzliya (IDC) ethics committee, with written informed consent from all subjects, in accordance with the Declaration of Helsinki. These studies protocols were approved by the IDC ethics committee.

## Results

### Stage One: Exploratory Factor Analysis (EFA)

**FIGURE 1:**
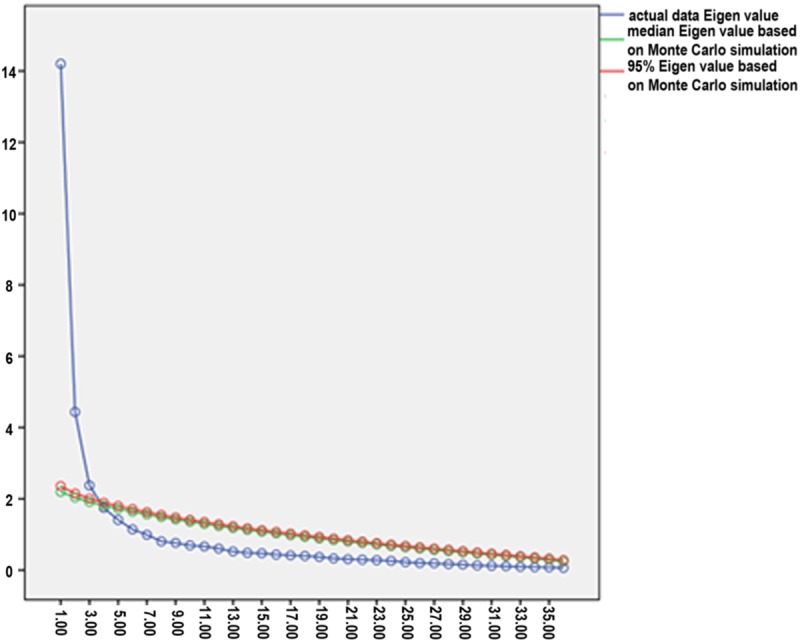
**Scree plot with parallel analysis results of 50% and 95% Eigen values, based on Monte Carlo Simulation**.

The first step was to examine the number of factors, or motivations, to produce a selfie. Both Cattell’s scree plot and parallel analysis (based on Humphreys and Montanelli’s1975 method, and on O’Connor’s2000 program) revealed a three factors model (see **Figure 1**). Based on this, a three factors model was examined, using a varimax rotation solution. A cut-off of 0.3 was used as a reference to indicate salient item loadings. The model accounted for 58.36% of the variance in selfie motivations scores. It should be noted that other two competing models, of one-factor and two-factor solutions, were also examined using a varimax rotation solution and a cut-off of 0.3. These models arouse a weaker amount of explained variance (one-factor solution: 39.45%, two-factors solution: 51.77%), supporting the scree plot and parallel analysis three factors solution.

Thus, we remain with the three-factors solution. Items that were load over more than one-factor removed from the final model. Leaving the final solution with 17 items, loaded upon three-factors. The EFA three-factors solution can be seen in **Table 1**.

**Table 1:** Rotated exploratory factor loadings of selfie motivations, using sample 1 data.

I’m taking selfies because…	Item #	Factor 1: self-approve	Factor 2: belonging	Factor 3: documentation
It builds my self-confidence	1	0.778		
It makes me look at myself in a positive way	2	0.768		
It might make me perceived as attractive to the opposite sex	3	0.717		
It is a way for me to receive love	4	0.691		
I must do it to feel good with myself	5	0.653		
My friends upload selfies, so I’m uploading too	6		0.817	
It makes me feel part of the society	7		0.808	
It makes me feel a strong feeling of belonging	8		0.777	
I don’t want to be the only one who not takes selfies	9		0.753	
This is the current trend, and everyone are doing it	10		0.752	
I want to be like everyone	11		0.751	
It supplies documentation for my experiences	12			0.853
It is a good and easy way to preserve memories	13			0.795
It allows me to remember places and experiences that I’ve experienced	14			0.783
I want to perpetuate the moment from my own point of view	15			0.769
I want a souvenir from places that I visited	16			0.764
I want others to see what I’ve experienced	17			0.550


The first factor, relates to the motivation to take selfie to confirm inner feelings, needs, and believes, was labeled “self-approval.” The second factor, discusses the motivation to take selfie to feel part of a group and to obey social morns, was labeled “belonging.” The third factor, talks about the motivation to keep memories from one’s own point of view, was labeled “documentation.”

### Stage Two: Confirmatory Factor Analysis (CFA)

**FIGURE 2:**
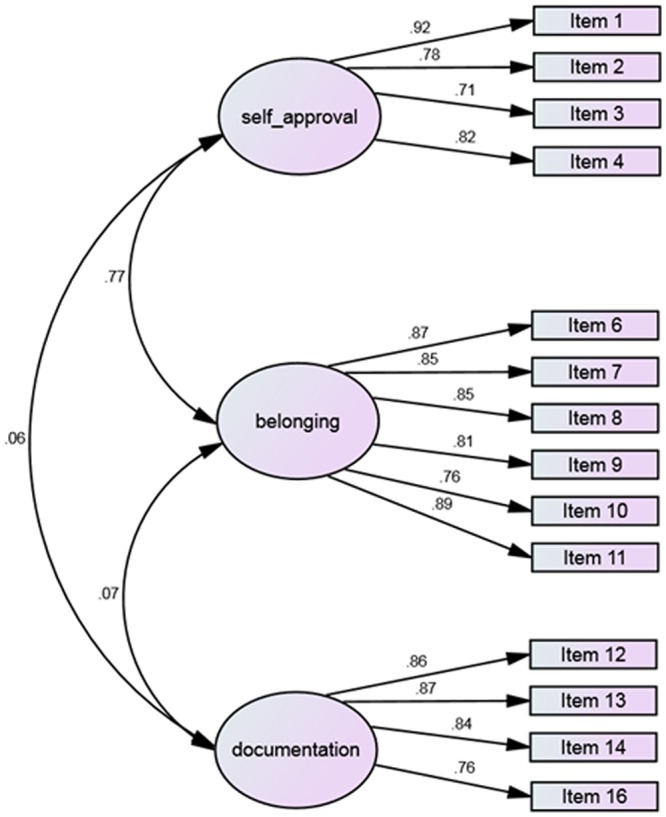
**The path diagram of standardized estimates of three selfie motivations, based on sample 2 data**.

Using the second dataset, CFA was conducted in AMOS software, to examine the model fit. The model suggested mediocre fitting (*X*^2^/*df* = 3.03, CFI = 0.91, GFI = 0.81, TLI = 0.89, SRMR = 0.11, RMSEA = 0.10). In the light of this mediocre model fit, we examine the standardized residual co-variances, and three items, that their standardized residual co-variances were higher than 2, excluded from the model. These items were: “I must do it to feel good with myself” (item number 5), “I want to perpetuate the moment from my own point of view” (item number 15), and “I want others to see what I’ve experienced” (item number 17). Removing these items from the model emerge a good model fit, confirming that the hypothesized factors fit well (*X*^2^/*df* = 2.02, CFI = 0.96, GFI = 0.90, TLI = 0.95, SRMR = 0.05, RMSEA = 0.07). The path diagram of standardized estimates was illustrated in **Figure 2**.

#### Comparison with Two-Factor Model

Following a reviewer’s suggestion, we combined “self-approve” and “belonging” factors, which were highly correlated, and examined a two-factor model. Here as well, there were three items with standardized residual co-variances that were higher than 2, we excluded from the model. The items were: “It allows me to remember places and experiences that I’ve experienced” (item number 14), “I want to perpetuate the moment from my own point of view” (item number 15), and “I want others to see what I’ve experienced” (item number 17). The model fit was lower than the three-factor model above any fitting index (X^2^/*df* = 4.49, CFI = 0.87, GFI = 0.768, TLI = 0.84, SRMR = 0.06, RMSEA = 0.13). Relative measurements to model comparisons (Penny, 2012), AIC and BIC, were also both lower in the three-factor model (AIC = 211.511, BIC = 318.662) compared with the two-factor model (AIC = 399.32, BIC = 493.49), giving a more direct indication that the three-factor model has a better fit as compared with the two-factor model.

#### Reliabilities

The cronbach’s α coefficient for the self-approving scale was 0.88, for the belonging scale was 0.93, and for the documentation scale 0.90, indicating a good reliability for all three scales.

#### Analyses of Excluded Items

The first item list included 35 items, while in the final selfie motivation questionnaire, only 14 questions remained. To examine whether the excluded items contained any further information that was missing from the final questionnaire, we created an additional EFA, similar to the first one, based on the items that were collected in Study 1, which contains all the initial collected items. We used similar methods to those used in Study 1, with a varimax rotation solution and a cut-off of 0.3 as a reference to indicate salient item loading. Cattell’s scree plot was offered as a three-factors solution. The items that remain after cleaning items that were load over more than one-factor are presented in **Table 2**. These three-factors shared a similar meaning with the three-factors that are part of the selfie questionnaire, indicating that by excluding these items from the final questionnaire, we did not miss any further information.

**Table 2:** Rotated exploratory factor loadings of excluded selfie motivations, using sample 1 data.

*I’m taking selfies because…*	Factor 1	Factor 2	Factor 3
This is how you meet new and interesting people	0.837		
It helps me widen my social circle	0.796		
I believe it’s one of the best ways to meet new people	0.781		
It opens opportunities for me to meet new people	0.757		
It helps me build relationships with others	0.686		
I must do it to feel good with myself	0.676		
It allows me to be seen at my best and to be perceived as popular in other people’s eyes		0.792	
I want others to see what I’ve experienced			0.822
Because it’s fun to share others in my daily experience			0.700
Because I want to publish whatever I’m doing			0.648
I want to perpetuate the moment from my own point of view			0.631


### Different Selfie Motivation as Connected to Personality Dimensions

Based on the data from sample 2, we also examine whether the three distinct selfie motivations are correlated with different personality characteristics. **Table 3** presents the correlations between the motivations and the personality traits.

**Table 3:** Means, standard deviations, and correlation matrix for all variables, based on sample 2 data.

	*M*	*SD*	1	2	3	4	5	6	7	8	9	10	11	12	13
(1) Self-approve motivation	1.696	1.08	1												
(2) Belonging motivation	1.69	0.935	0.715ˆ**	1											
(3). Documentation motivation	3.39	1.20	0.047	0.072	1										
(4) Uploading selfies frequency	3.79	0.549	-0.216ˆ**	-0.154ˆ*	-0.148ˆ*	1									
(5) Checking “likes” frequency	3.67	1.15	-0.291ˆ**	-0.081	-0.050	0.244ˆ**	1								
(6) Narcissism	3.18	0.593	-0.013	-0.028	0.066	0.011	-0.130	1							
(7) Self-esteem	32.49	5.41	-0.153ˆ*	-0.125	0.129	-0.004	-0.017	0.337ˆ**	1						
(8) Extraversion	4.56	1.16	-0.142	-0.109	0.227ˆ**	-0.103	-0.086	0.207ˆ*	0.273ˆ**	1					
(9) Agreeableness	4.83	1.08	-0.051	0.010	0.161ˆ*	-0.077	0.092	-0.139	0.196ˆ*	0.067	1				
(10) Conscientiousness	5.25	1.38	-0.201ˆ*	-0.117	0.046	0.110	0.041	0.135	0.165ˆ*	0.200ˆ*	0.082	1			
(11) Emotional Stability	4.13	1.41	-0.200ˆ*	-0.022	0.068	0.092	0.061	0.155	0.370ˆ**	0.180ˆ*	0.232ˆ**	0.128	1		
(12) Openness to experiences	4.78	1.32	-0.183ˆ*	-0.235ˆ**	0.030	-0.103	0.238ˆ**	0.196ˆ*	0.144	-0.006	-0.025	-0.127	0.127	1	
(13) Gender	1.67	0.471	0.031	-0.033	0.274ˆ**	-0.101	-0.149ˆ*	-0.202ˆ*	-0.031	0.110	0.137	0.071	-0.103	-0.183ˆ*	1


*M, Mean; SD, Standard Deviation, ^∗^p < 0.05 level, ^∗∗^p < 0.01 level*.

#### Selfie Behaviors

Not surprisingly, all three motives were related to frequency of selfie taking, suggesting that higher selfie motivations are related to greater frequency of selfie posting (self-approve: *r* = -0.22, *p* < 0.01, belonging: *r* = -0.15, *p* < 0.05, documentation: *r* = -0.15, *p* < 0.05). However, only self-approval motivation was related to frequency of likes checking (*r* = -0.292, *p* < 0.001), indicating that self-approval motivation arise liking checking.

#### Narcissism

Unlike other studies, our results did not support the idea that selfie motivations are related to narcissism. None of the three selfie motivations were related to narcissism (self-approve: *r* = -0.013, *p* = 0.87; belonging: *r* = -0.028, *p* = 0.72; documentation: *r* = 0.07, *p* = 0.41). Since evidence suggests that selfies are related to narcissism in men but not in women (Sorokowski et al., 2015), we examine the relationship between the three selfie motivations and narcissism separately to each gender, however, could not find any significant effect (for men, all *p*’s > 0.33; for women, all *p*’s > 0.15). Moreover, we conducted three regression analyses in steps, to examine whether gender has a moderating role in the connection between narcissism and each of the three selfie motivations. In the first step of each regression, gender and narcissism were entered. In the second step, the multiplication of gender and narcissism was entered. As a dependent variable, we used one selfie motivation at each regression analysis. None of these regressions were found a significant interaction between gender and narcissism in their ability to predict selfie motivation (self-approve: β = 0.027, *p* = 0.96, belonging: β = 0.58, *p* = 0.30, documentation: β = -0.001, *p* = 0.99)

#### Self-esteem

Self-esteem was negatively correlated to self-approval motivation (*r* = -0.15, *p* < 0.05), and was not correlated to either belonging or documentation motivations.

#### Big Five

Big five traits were able to differentiate between the selfie motivations: documentation motivation, but not the other selfie motivations, was positively related to extroversion (*r* = 0.23, *p* < 0.005) and agreeableness (*r* = 0.16, *p* < 0.05). Self-approval motivation, but not the other motives, was negatively related to conscientiousness (*r* = -0.20, *p* < 0.05) and to emotional stability (*r* = -0.20, *p* < 0.05). Self-approval and belonging motivations were negatively related to openness to experiences (*r* = -0.18, *p* < 0.05; *r* = -0.24, *p* < 0.01, respectively).

## Discussion

The major aim of the study was to reveal the number and type of motivations behind the taking of selfies. As predicted, the EFA and the good fit of the CFA indicated that more than one motivation might instigate selfie taking. The factor analyses displayed three distinct selfie motivations: self-approval, belonging and documentation. Self-approval is the need to validate one’s confidence or significance by taking selfies. Belonging is the tendency to take and upload selfies and obeying the social norms, in order to feel part of one’s environment. Documentation is the intention to preserve one;s memory and experience by taking a selfie.

Moreover, we suggested that if several selfie motivations were revealed, each motivation would be differently connected to different personality traits. Results indicate that mostly, this was the case. But, it should be taken into account that some of these relationships were weak, indicating a limited effect sizes. However, although these connections might be smaller than excepted, all of them are perceived as reasonable in the light of pervious research, which showed similar trends.

Self-approval motivation was negatively related to conscientiousness. Conscientiousness is one of the big five personality traits and relates to persistence, self-oriented motivations, and a decreased need to conform (John and Srivastava, 1999; Roccas et al., 2002). Our finding, suggests that it also relates to a higher need to seek your self-confirmation from an external source as selfies, seems to continue the same line of findings. Self-approval motivation was negatively related to emotional stability, implying that gaining your own confidence from others’ opinions is not indicative of a stable emotional state. A similar interpretation can be given to the negative relationship between the motives of self-esteem and self-approval: if one seeks confidence and approval from others, then one’s self-esteem is dependent upon their opinion. This reinforces an earlier finding that people perceive their self-esteem as higher in areas that relate to characteristics that others believe they possess (MacDonald et al., 2003). Moreover, having conscientiousness, emotional stability and self-esteem negatively relate to self-approval may also explain the positive correlation between this motive and the frequent need to check for “likes” after posting a selfie on SNS. When someone’s self-worth is not determined by their own inner compass, it is no wonder that s/he is searching for external reinforcements, in this case, in the shape of a “like.”

Both motivators of self-approval and belonging were negatively related to openness to experiences. A high score in openness to experiences is related to higher risk taking (Nicholson et al., 2005), higher creativity (George and Zhou, 2001), and higher self- directed values (Roccas et al., 2002). These motives both have roots that relate to trying to be normative and conventional, in the self-approval motive, for your own confirmation, and in the belonging motive, in order to fit to the social norms. As openness to experience is somewhat the opposite of being normative (McCrae, 1987, 1993), these two motives are negatively correlated with it.

Aside from openness to experiences, belonging was not related to any other characteristic. It is surprising, as belonging is one of the basic needs (Maslow, 1971), that would predicted to be connected to some personality variables. We suggest that as belonging, in this research, is the motive to be socially normative, none of our personality measures may be relevant to this motive: none of them is a measurement of conformity or importance of social norms. Future studies should examine the role of belonging as a motivator in other personality traits, such as conformity and locus of control. Ever since Asch’s famous study, it is clear that people who are higher on the need to belong will also conform to the group, even at the cost of lying, but this will not be the case for those who have a lower need to belong (Asch, 1956, see also: Turner, 1987; Adler et al., 1992; Harris, 1995). Similarly, we predict that a higher need for belonging will be related to a greater external locus of control, as external locus of control means gaining control from others, and not from the inner self (Biondo and MacDonald, 1971; Lefcourt, 1976), which in today’s world may come to fruition through the taking of selfies because everyone else is doing so.

Documentation was positively related to both extraversion and agreeableness. Agreeableness might be explained as a reason behind the type of selfie taking. As Paris and Pietschnig (2015) demonstrated, travel selfies are related to agreeableness, which might imply that this personality trait influences the type of activity one is doing, and this activity type leads to different type of selfie motives. This may well be the case for extraversion, since extraverts are higher on hedonism and stimulation (Roccas et al., 2002), when they take an excursion they are likely to take travel selfies for the purpose of documenting their experiences. Further study should examine the type of selfies that are common for each motive, and examine whether the selfie-type is mediated between personality traits and selfie motives.

Unlike other studies, none of the selfie motives were found to be related to narcissism. Even when, following the earlier study (Sorokowski et al., 2015), we examined this relationship as divided by gender, or when we used gender as a moderator. A possible explanation to this gap might be found in previous studies about the relationship between selfies and narcissism, since some of these did not find a straightforward connection between narcissism and selfie behaviors (for example: Barry et al., 2015; Sorokowski et al., 2015). However, there is evidence that several specific dimensions of narcissism and not narcissism in general, are related to selfie behaviors. Specifically, it was found that grandiose exhibitionism facets and vulnerable narcissism are related to selfie posting (Barry et al., 2015; Weiser, 2015). Future studies should examine whether specific narcissism dimensions are also related to the three different selfie motives that were found in this study. It is reasonable to assume, for example, that the self-approval motive is related to vulnerable narcissism, as they both relate to maintaining self-confirmation using external cues.

Another explanation for the gap between the literature and our study might be that studies about narcissism-selfie connections mostly focus on posting selfies to SNS, whereas in our study, we asked our participants to refer to selfie taking. As mentioned, most of the selfies are in fact not uploaded to SNS (Katz and Crocker, 2015), therefore there should be a distinction made between taking and posting selfies. Narcissists, who tend to be attention seekers and exhibitionists (Vazire et al., 2008), might give additional weight to posting selfies to SNS, which is a public act, and less importance to selfie taking in general, as it is a private behavior. In such case, it is reasonable that our research, focusing on selfie taking, did not find a connection between the taking of selfies and narcissism, that was present in other studies which focused on selfie posting. We suggest that future studies on the role of narcissism in selfies should study the differences between the private action of taking selfies, and the public practice of posting selfies to SNS.

This research suffers from several limitations: first, participants in both research stages were undergraduate students. As social network behaviors change with age (Carrier et al., 2009; Turner, 2015), and specifically as selfie behaviors change with age (Dhir et al., 2016), these results represent only a certain population. Secondly, this study is based on self-reported data. The relationships between personality traits and SNS behaviors have been shown to be different when measured by self-reporting or by more objective criteria (Amichai-Hamburger and Vinitzky, 2010). Thus, the results of the current study should be interpreted only as part of greater picture, that also includes selfie behaviors and their motivational meanings.

## Conclusion

This research served as initial foray into the search for different selfie motivations. It indicates three main motives behind the taking of selfies, and their ability to distinct between different personality traits. Since selfie taking is such a huge phenomenon, we perceive this research as a primary exploration that should be extended to ensure a deeper understanding of the different selfie motives and their implications for on individuals, as well as for society.

## Author Contributions

YA-H developed the study concept; SE and YA-H collected the data; SE analyzed and interpreted the data; and SE and YA-H wrote and revised the manuscript.

## Conflict of Interest Statement

The authors declare that the research was conducted in the absence of any commercial or financial relationships that could be construed as a potential conflict of interest.

## References

[B1] Adler P. A. Kless S. J. Adler P. (1992). Socialization to gender roles: popularity among elementary school boys and girls. *Sociol. Educ.* 65 169–187. 10.2307/2112807

[B2] Ames D. R. Rose P. Anderson C. P. (2006). The NPI-16 as a short measure of narcissism. *J. Res. Pers.* 40 440–450. 10.1037/a0033192

[B3] Amichai-Hamburger Y. Vinitzky G. (2010). Social network use and personality. *Comput. Hum. Behav.* 26 1289–1295. 10.1016/j.chb.2010.03.018

[B4] Asch S. E. (1956). Studies of independence and conformity: I. A minority of one against a unanimous majority. *Psychol. Monogr. Gen. Appl.* 70 1–70. 10.1037/h0093718

[B5] Barry C. T. Doucette H. Loflin D. C. Rivera-Hudson N. Herrington L. L. (2015). “Let me take a selfie”: associations between self-photography, narcissism, and self-esteem. *Psychol. Pop. Media Cult.* 6 48–60. 10.1037/ppm0000089

[B6] Biondo J. MacDonald A. P. Jr. (1971). Internal-external locus of control and response to influence attempts. *J. Pers.* 39 407–419. 10.1111/j.1467-6494.1971.tb00051.x 5113652

[B7] Carrier L. M. Cheever N. A. Rosen L. D. Benitez S. Chang J. (2009). Multitasking across generations: multitasking choices and difficulty ratings in three generations of Americans. *Comput. Hum. Behav.* 25 483–489. 10.1016/j.chb.2008.10.012

[B8] Dhir A. Pallesen S. Torsheim T. Andreassen C. S. (2016). Do age and gender differences exist in selfie-related behaviours? *Comput. Hum. Behav.* 63 549–555. 10.1016/j.chb.2016.05.053

[B9] George J. M. Zhou J. (2001). When openness to experience and conscientiousness are related to creative behavior: an interactional approach. *J. Appl. Psychol.* 86 513–524. 10.1037/0021-9010.86.3.513 11419810

[B10] Gonzales A. L. Hancock J. T. (2011). Mirror, mirror on my facebook wall: effects of exposure to facebook on self-esteem. *Cyberpsychol. Behav. Soc. Network.* 14 79–83. 10.1089/cyber.2009.0411 21329447

[B11] Gosling S. D. Rentfrow P. J. Swann W. B. (2003). A very brief measure of the Big-Five personality domains. *J. Res. Pers.* 37 504–528. 10.1016/S0092-6566(03)00046-1

[B12] Halpern D. Valenzuela S. Katz J. E. (2016). “Selfie-ists” or “Narci-selfiers”?: a cross-lagged panel analysis of selfie taking and narcissism. *Pers. Ind. Dif.* 97 98–101. 10.1016/j.paid.2016.03.019

[B13] Hamid N. A. Ishak M. S. Yazam S. S. N. M. (2015). Facebook, youtube and instagram: exploring their effects on undergraduate students’ personality traits. *J. Soc. Med. Soc.* 4 138–165.

[B14] Harris J. R. (1995). Where is the child’s environment? A group socialization theory of development. *Psychol. Rev.* 102 458–489. 10.1037/0033-295X.102.3.458

[B15] Hsiao K. L. Shu Y. Huang T. C. (2017). Exploring the effect of compulsive social app usage on technostress and academic performance: perspectives from personality traits. *Telemat. Inform.* 34 679–690. 10.1016/j.tele.2016.11.001

[B16] Humphreys L. G. Montanelli R. G. Jr. (1975). An investigation of the parallel analysis criterion for determining the number of common factors. *Multiv. Behav. Res.* 10 193–205. 10.1207/s15327906mbr1002_5

[B17] Jain A. Gera N. Ilavarasan P. V. (2016). Whether social media use differs across different personality types? Insights for managing human resources. *Int. J. Work Organ. Emot.* 7 241–256. 10.1504/IJWOE.2016.081465

[B18] John O. P. Srivastava S. (1999). The big five trait taxonomy: history, measurement, and theoretical perspectives. *Handb. Pers. Theory Res.* 2 102–138.

[B19] Katz J. E. Crocker E. T. (2015). Selfies and photo messaging as visual conversation: reports from the United States, United Kingdom and China. *Int. J. Commun.* 9 1861–1872.

[B20] Lee J. A. Sung Y. (2016). Hide-and-seek: narcissism and “Selfie”-related behavior. *Cyberpsychol. Behav. Soc. Network.* 19 347–351. 10.1089/cyber.2015.0486 27028460

[B21] Lefcourt H. M. (1976). *Locus of Control.* New York, NY: Lawrence Erlbaum Associates.

[B22] MacDonald G. Saltzman J. L. Leary M. R. (2003). Social approval and trait self-esteem. *J. Res. Pers.* 37 23–40. 10.1016/S0092-6566(02)00531-7

[B23] Marshall T. C. Lefringhausen K. Ferenczi N. (2015). The Big Five, self-esteem, and narcissism as predictors of the topics people write about in Facebook status updates. *Pers. Individ. Dif.* 85 35–40. 10.1016/j.paid.2015.04.039

[B24] Maslow A. H. (1971). *Farther Reaches of Human Nature.* New York, NY: Viking Press.

[B25] McCrae R. (1987). Creativity, divergent thinking, and openness to experience. *J. Pers. Soc. Psychol.* 52 1258–1265. 10.1037/0022-3514.52.6.1258

[B26] McCrae R. R. (1993). Openness to experience as a basic dimension of personality. *Imagin. Cogn. Pers.* 13 39–55. 10.2190/H8H6-QYKR-KEU8-GAQ0

[B27] Moon J. H. Lee E. Lee J. A. Choi T. R. Sung Y. (2016). The role of narcissism in self-promotion on instagram. *Pers. Individ. Dif.* 101 22–25. 10.1016/j.paid.2016.05.042

[B28] Nicholson N. Soane E. Fenton-O’Creevy M. Willman P. (2005). Personality and domain-specific risk taking. *J. Risk Res.* 8 157–176. 10.1080/1366987032000123856

[B29] O’Connor B. P. (2000). SPSS and SAS programs for determining the number of components using parallel analysis and Velicer’s MAP test. *Behav. Res. Methods Instrum. Comput.* 32 396–402. 10.3758/BF03200807 11029811

[B30] Oxford dictionaries (2013). *The Oxford Dictionaries Word of the Year 2013.* Available at: http://blog.oxforddictionaries.com/press-releases/oxford-dictionaries-word-of-the-year-2013/

[B31] Paris C. M. Pietschnig J. (2015). *‘But First, Let Me Take a Selfie’: Personality Traits as Predictors of Travel Selfie Taking and Sharing Behaviors.* Available at: http://scholarworks.umass.edu/ttra/ttra2015/Academic_Papers_Oral/1

[B32] Penny W. D. (2012). Comparing dynamic causal models using AIC, BIC and free energy. *Neuroimage* 59 319–330. 10.1016/j.neuroimage.2011.07.039 21864690 PMC3200437

[B33] Roccas S. Sagiv L. Schwartz S. H. Knafo A. (2002). The big five personality factors and personal values. *Pers. Soc. Psychol. Bull.* 28 789–801. 10.1177/0146167202289008

[B34] Rosenberg M. (1965). Rosenberg Self-Esteem Scale (RSE). *Acceptance and Commitment Therapy. Measures package.*

[B35] Ross C. Orr E. S. Sisic M. Arseneault J. M. Simmering M. G. Orr R. R. (2009). Personality and motivations associated with Facebook use. *Comput. Hum. Behav.* 25 578–586. 10.1016/j.chb.2008.12.024

[B36] Ryan T. Xenos S. (2011). Who uses Facebook? An investigation into the relationship between the Big Five, shyness, narcissism, loneliness, and Facebook usage. *Comput. Hum. Behav.* 27 1658–1664. 10.1016/j.chb.2011.02.004

[B37] Seidman G. (2013). Self-presentation and belonging on Facebook: how personality influences social media use and motivations. *Pers. Individ. Dif.* 54 402–407. 10.1016/j.paid.2012.10.009

[B38] Sheldon P. Bryant K. (2016). Instagram: motives for its use and relationship to narcissism and contextual age. *Comput. Hum. Behav.* 58 89–97. 10.1016/j.chb.2015.12.059

[B39] Sorokowski P. Sorokowska A. Oleszkiewicz A. Frackowiak T. Huk A. Pisanski K. (2015). Selfie posting behaviors are associated with narcissism among men. *Pers. Individ. Dif.* 85 123–127. 10.1016/j.paid.2015.05.004

[B40] Turner A. (2015). Generation Z: technology and social interest. *J. Individ. Psychol.* 71 103–113. 10.1353/jip.2015.0021

[B41] Turner J. C. (1987). *Rediscovering the Social Group: A Self-Categorization Theory.* Oxford: Basil Blackwell.

[B42] Vazire S. Naumann L. P. Rentfrow P. J. Gosling S. D. (2008). Portrait of a narcissist: manifestations of narcissism in physical appearance. *J. Res. Pers.* 42 1439–1447. 10.1016/j.jrp.2008.06.007

[B43] Warfield K. (2014). *Making Selfies/Making Self: Digital Subjectivities in the Selfie.* Available at: http://kora.kpu.ca/facultypub/8

[B44] Weiser E. B. (2015). #Me: narcissism and its facets as predictors of selfie-posting frequency. *Pers. Individ. Dif.* 86 477–481. 10.1016/j.paid.2015.07.007

[B45] Wickel T. M. (2015). Narcissism and social networking sites: the act of taking selfies. *Elon J. Undergrad. Res. Commun.* 6 5–12.

